# The entropic brain: a theory of conscious states informed by neuroimaging research with psychedelic drugs

**DOI:** 10.3389/fnhum.2014.00020

**Published:** 2014-02-03

**Authors:** Robin L. Carhart-Harris, Robert Leech, Peter J. Hellyer, Murray Shanahan, Amanda Feilding, Enzo Tagliazucchi, Dante R. Chialvo, David Nutt

**Affiliations:** ^1^Division of Brain Sciences, Department of Medicine, Centre for Neuropsychopharmacology, Imperial College LondonLondon, UK; ^2^C3NL, Division of Brain Sciences, Department of Medicine, Imperial College LondonLondon, UK; ^3^Department of Computing, Imperial College LondonLondon, UK; ^4^The Beckley Foundation, Beckley ParkOxford, UK; ^5^Neurology Department and Brain Imaging Center, Goethe UniversityFrankfurt am Main, Germany; ^6^Consejo Nacional de Investigaciones Científicas y Tecnológicas (CONICET)Buenos Aires, Argentina

**Keywords:** serotonin, default mode network, criticality, entropy, 5-HT2A receptor, metastability, consciousness, REM sleep

## Abstract

Entropy is a dimensionless quantity that is used for measuring uncertainty about the state of a system but it can also imply physical qualities, where high entropy is synonymous with high disorder. Entropy is applied here in the context of states of consciousness and their associated neurodynamics, with a particular focus on the psychedelic state. The psychedelic state is considered an exemplar of a primitive or primary state of consciousness that preceded the development of modern, adult, human, normal waking consciousness. Based on neuroimaging data with psilocybin, a classic psychedelic drug, it is argued that the defining feature of “primary states” is elevated entropy in certain aspects of brain function, such as the repertoire of functional connectivity motifs that form and fragment across time. Indeed, since there is a greater repertoire of connectivity motifs in the psychedelic state than in normal waking consciousness, this implies that primary states may exhibit “criticality,” i.e., the property of being poised at a “critical” point in a transition zone between order and disorder where certain phenomena such as power-law scaling appear. Moreover, if primary states are critical, then this suggests that entropy is suppressed in normal waking consciousness, meaning that the brain operates just below criticality. It is argued that this entropy suppression furnishes normal waking consciousness with a constrained quality and associated metacognitive functions, including reality-testing and self-awareness. It is also proposed that entry into primary states depends on a collapse of the normally highly organized activity within the default-mode network (DMN) and a decoupling between the DMN and the medial temporal lobes (which are normally significantly coupled). These hypotheses can be tested by examining brain activity and associated cognition in other candidate primary states such as rapid eye movement (REM) sleep and early psychosis and comparing these with non-primary states such as normal waking consciousness and the anaesthetized state.

## Introduction

The main aim of this paper is to introduce a new theory of conscious states that incorporates principles of physics, neurobiology, and psychoanalysis. The theory is intended to assist our understanding of the makeup of the human mind, addressing such questions as: “how does the normal waking consciousness of healthy adult humans relate to other states of consciousness?” “how does the human brain maintain its normal state of waking consciousness?” and “what happens to the human brain's functionality when non-ordinary states such as rapid eye movement (REM) sleep/dreaming, early psychosis and the psychedelic state occur?”

At its core, the entropic brain hypothesis proposes that the quality of any conscious state depends on the system's entropy^1^ measured via key parameters of brain function. Entropy is a powerful explanatory tool for cognitive neuroscience since it provides a quantitative index of a dynamic system's randomness or disorder while simultaneously describing its informational character, i.e., our uncertainty about the system's state if we were to sample it at any given time-point. When applied in the context of the brain, this allows us to make a translation between mechanistic and qualitative properties. Thus, according to this principle, increased subjective uncertainty or “puzzlement” accompanies states of increased system entropy. These ideas are consistent with Karl Friston's free-energy principle^2^ and readers interested in Bayesian inference and the mechanisms by which the brain is hypothesized to minimize free-energy/surprise should consult this work (Friston, 2010).

System entropy, as it is applied to the brain, is related to another current hot-topic in cognitive neuroscience, namely “self-organized criticality”^3^ (Chialvo et al., 2007). The phenomenon of self-organized criticality refers to how a complex system (i.e., a system with many constituting units that displays emergent properties at the global-level beyond those implicated by its individual units) forced away from equilibrium by a regular input of energy, begins to exhibit interesting properties once it reaches a critical point in a relatively narrow transition zone between the two extremes of system order and chaos. Three properties displayed by critical systems that are especially relevant to the present paper are: (1) a maximum number of “metastable” or transiently-stable states (Tognoli and Kelso, 2014), (2) maximum sensitivity to perturbation, and (3) a propensity for cascade-like processes that propagate throughout the system, referred to as “avalanches” (Beggs and Plenz, 2003). There is growing evidence that brain activity, like much of nature, displays critical behavior (Beggs and Plenz, 2003)—and this raises some interesting questions: e.g., does the brain activity of healthy-adult-humans exhibit characteristics of criticality during normal waking consciousness, or are there other states of consciousness in which these characteristics are even more pronounced?

Another major topic that is covered in this paper is the psychoanalytic model of the structure of the mind (i.e., Freud's “metapsychology”). Specifically, we discuss some of the most fundamental concepts of Freudian metapsychology, with a special focus on the ego^4^. We focus on the ego because it is one of Freud's less abstract constructs and it is hypothesized that its disintegration is necessary for the occurrence of primary states. The ego can be defined as a sensation of possessing an immutable identity or personality; most simply, the ego is our “sense of self.” Importantly however, in Freudian metapsychology, the ego is not just a (high-level) sensation of self-hood; it is a fundamental system that works in competition and cooperation with other processes in the mind to determine the quality of consciousness. It is because Freud described “the ego” in this mechanistic sense that it can be considered a useful complement to the more widely used concept of “the self.” Effectively, the terms “ego” and “self” are synonyms, except that “the ego” has a background in Freudian metapsychology.

Finally, the shared topic that connects all of the above and offers a unique potential for their empirical study is the psychedelic drug state. In the following section we make the case that scientific research with psychedelics has considerable potential for developing aspects of psychoanalytic theory and for studying human consciousness more generally. Citing recent neuroimaging findings involving the classic psychedelic drug, psilocybin, the psychedelic state is described as a prototypical high-entropy state of consciousness (i.e., higher than normal waking consciousness). Intriguingly, we show evidence that the brain exhibits more characteristics of criticality in the psychedelic state than are apparent during normal waking consciousness. Moreover, this leads to the proposal that the brain of modern adult humans differs from that of its *closest* evolutionary and developmental antecedents because of an extended capacity for entropy suppression, implying that the system (i.e., the brain) gravitates away from criticality proper toward a state of slight sub-criticality. The psychological counterpart of this process is the development of a mature ego^5^ and associated metacognitive functions (see below for relevant definitions of these terms). Specifically, we propose that within-default-mode network (DMN)^6^ resting-state functional connectivity (RSFC)^7^ and spontaneous, synchronous oscillatory activity in the posterior cingulate cortex (PCC), particularly in the alpha (8–13 Hz) frequency band, can be treated as neural correlates of “ego integrity.” Evidence supporting these hypotheses is discussed in the forthcoming sections.

Before beginning it is important to address an initial point of potential ambiguity. The view taken here is that the human brain exhibits greater entropy than other members of the animal kingdom, which is equivalent to saying that the human mind possesses a greater repertoire of potential mental states than lower animals (see Giulio Tononi's information integration theory of consciousness cited below). Thus, if referring to human evolution beyond our closest *surviving* relatives then it would be misleading to suggest that entropy-suppression is the defining property of the human brain—indeed, it might be more accurate to speak of entropy-expansion. The evolution of human consciousness may have occurred through a process of relatively rapid entropy-expansion (with a concomitant increase in system disorder) followed by entropy-suppression (or system re-organization and settling). Thus, the proposal that normal waking consciousness in healthy, adult, modern humans depends on entropy suppression implies that there was a state relatively proximal to this (e.g., in archaic homo-sapiens and in infants) in which entropy was relatively elevated, as it is in primary states. The point is that the brain of adult modern-humans is in a settling rather than expanding phase.

## The research value of psychedelics

“It does not seem to be an exaggeration to say that psychedelics, used responsibly and with proper caution, would be for psychiatry what the microscope is for biology and medicine or the telescope is for astronomy. These tools make it possible to study important processes that under normal circumstances are not available for direct observation.” (Grof, 1980)

In 1953, the British research psychiatrist Humphrey Osmond was investigating the psychotomimetic (psychosis mimicking) effects of mescaline, a psychedelic drug derived from the peyote cactus. The British author Aldous Huxley learned of Osmond's work and struck up a correspondence, requesting that Osmond supervise a personal psychedelic experience. Huxley's subsequent mescaline experience would become the subject of his famous book “*The Doors of Perception*” (Huxley, 1954). Like many before and after him, Huxley was profoundly affected by his experiences with psychedelics and in 1956 sought with Osmond a satisfactory term for this class of drugs. At the time, “psychotomimetics” and “hallucinogens” were popular, but both men felt that these referred to mere aspects of the drug experience and not its essential character. Huxley suggested “phanerothyme,” intending to mean “bringing forth the spirit or soul” (Huxley et al., 1977), and Osmond offered “psychedelic” combining the Greek words for “mind” or “soul” (psychē) with “dclôsē,” meaning “to manifest.” While it was Osmond's “psychedelic” that would stick, it is telling that both men were searching for a word that could denote the same essential property, i.e., psychedelic's ability to make manifest latent aspects of the mind.

In 1943, Swiss chemist Albert Hofmann discovered the extraordinary psychological properties of lysergic acid diethylamide (LSD) (Hofmann, 1980) and the first reports on its effects appeared in scientific journals in the late 1940s. These papers immediately highlighted LSD's potential to be psychologically agitative. The first English language publication was released in 1950 and here the authors reported: “*the effect of LSD was a transitory toxic state, disturbing the barrier of repression and permitting a re-examination of significant experiences of the past that were sometimes relived with a frightening realism*.” (Busch and Johnson, 1950) In the following years, psychedelics became one of the most researched classes of psychoactive drug in science, with several hundred relevant publications (Grinspoon and Bakalar, 1979). During these years, the focus shifted from psychedelics as psychotomimetics to psychedelics as psychotherapeutic adjuncts, with major international conferences on the topic (Grinspoon and Bakalar, 1979) and even the construction of purpose-built psychedelic treatment centers (Sandison, 2001). Political pressure in the late 1960s led to the illegalization of psychedelics and this had a significant negative impact on legitimate scientific research (Grinspoon and Bakalar, 1979; Lee and Shlain, 1985)—a problem that continues today (Nutt et al., 2013). Despite this however, there has been a resurgence of scientific interest in psychedelics in recent years (Vollenweider et al., 1998; Nichols, 2004; Griffiths et al., 2006, 2008; Moreno et al., 2006; Gonzalez-Maeso et al., 2007; Grob et al., 2011; Carhart-Harris et al., 2012a).

The dominant theoretical and therapeutic approach during the early era of psychedelic research was psychoanalytic. Psychedelics were used therapeutically under the rationale that they work to lower psychological defenses to allow personal conflicts to come to the fore that can then be worked through with a therapist (Cohen, 1972). A related model was that the relinquishment of “ego” enabled profound existential or “peak” experiences to occur that could have a lasting positive impact on behavior and outlook (Savage, 1962). Innumerable cases of apparent spontaneous insights about “self” or “nature” exist in the literature on psychedelics (Cattell, 1954; Sandison, 1954; Sandison and Whitelaw, 1957; Denber, 1958; Hausner and Dolezal, 1965; Torda, 1969; Cohen, 1972; Grof, 1982) and reports of “ego-dissolution” or “disintegration” are commonplace among those who have experienced the effects of these drugs (Carhart-Harris and Nutt, 2010; Carhart-Harris et al., 2012b). Some psychiatrists even believed that psychedelics could provide the necessary scientific evidence for major psychoanalytic hypotheses (Sandison, 1954; Cohen, 1972; Grof, 1982). For example, one enthused: “*The phenomenology of the psychodynamic experiences in LSD sessions is to a large extent in agreement with the basic concepts of classical psychoanalysis… Observations from LSD psychotherapy could be considered laboratory proof of the basic Freudian premises*.” (Grof, 1982).

**Figure 1:**
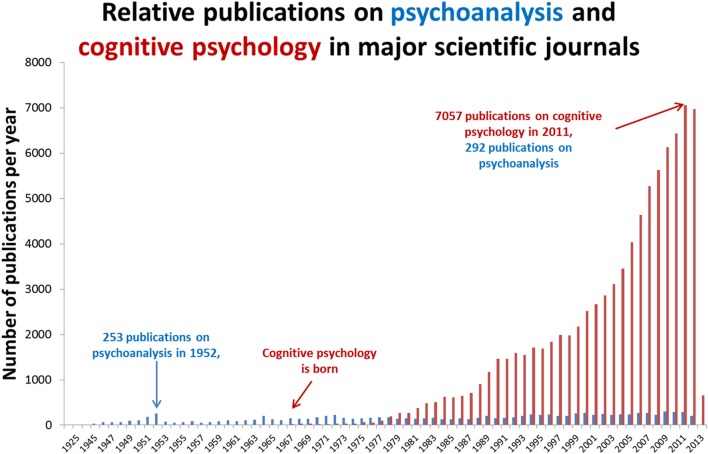
**Relative publications on psychoanalysis and cognitive psychology**. Annual publications in major medical science journals referenced in the leading database, PubMed. Articles were retrieved by entering the search terms “psychoanalysis” and “cognitive psychology” in separate searches using the default search parameters of PubMed. It is worth noting that psychoanalysis has a different publication culture to cognitive neuroscience. Articles on psychoanalysis are not always available via PubMed and many psychoanalytic writings are published in books rather than academic journals. With these caveats entered however, the publication count shown above still helps to illustrate the general point that psychoanalysis has failed to gain a significant foothold in mainstream analytical science.

Psychoanalytic theory dominated psychiatry in the 1950s but after influential critiques (Eysenck, 1973), the cognitive revolution (Neisser, 1967) and significant pharmacological developments in psychiatry (Ban, 2001a,b; Fink, 2010), its influence significantly waned. As illustrated in Figure 1, despite over a century since its inception, psychoanalysis has failed to establish itself as a science of the mind. This may be because its hypotheses are hollow (Webster, 1995) or because they do not easily lend themselves to controlled experiment. In contrast, cognitive psychology is a mechanistic framework for describing observable phenomena that has become the natural bedfellow for human neuroscience. In comparison with the spectacular success of cognitive psychology, what should we make of the relative stagnancy of psychoanalysis? Is psychoanalysis scientifically redundant? Its fiercest critics claim that it is a belief system, a tautology with untestable hypotheses (Webster, 1995) but others claim that it has considerable explanatory value but could benefit from a closer integration with cognitive neuroscience (Kandel, 1999; Carhart-Harris and Friston, 2010; Panksepp and Solms, 2012). The present article takes this latter view and argues that the most realistic way forward for psychoanalysis as a science is for its most tangible hypotheses to be simplified and applied within the framework of cognitive neuroscience. Here we take the view that this is a necessary concession for psychoanalysis if it is to develop its credibility as a model of the mind.

In what follows, a roadmap is presented for how scientific research with psychedelics can assist the integration of psychoanalysis with cognitive neuroscience in order to further our understanding of human consciousness. This is motivated by the view that psychoanalysis can contribute something substantial to the mind sciences because it bridges an explanatory gap that has been left vacant by cognitive psychology. This gap only exists because cognitive psychology (rightly) focuses on phenomena that can be observed and manipulated by controlled experiment but crucially, *without psychedelic drugs, it is virtually impossible to bring the core phenomena of psychoanalytic theory into an observable space.*

Freud famously said of dreams that they provide privileged access to the workings of the unconscious mind (Freud, 1937) but research on dreaming is fraught with difficulties because [despite the phenomenon of lucid dreaming (Ogilvie et al., 1982)] the dream experience cannot be easily reflected on and reported in real-time, and neither can its onset and offset be easily controlled. Thus, Freud's cherished “royal road” has not proved particularly regal and a more practical alternative is required if key psychoanalytic theories are to be incorporated into the mind sciences.

“If, as Freud said, dreams are the royal road to the unconscious, is it possible that psychedelic drugs are a superhighway to the unconscious?” (Holden, 1980)

This article argues that controlled studies with psychedelics are capable of providing major new insights into the nature of the mind and how it arises from brain activity. This is because the mind must be thoroughly deconstructed in order for us to become cognizant of its constituents and how they interact to give rise to global phenomena. The unique scientific value of psychedelics rests on their ability to selectively target processes that appear to be critical for the maintenance of normal waking consciousness. In addressing the action of psychedelic drugs on the brain, this article begins at the cellular level before progressing to the systems level. The intention is to offer a comprehensive account of how psychedelics alter brain function to alter consciousness.

Somewhat uniquely, psychedelics can be studied at a range of epistemological levels; from molecular pharmacology (Gonzalez-Maeso and Sealfon, 2009) to psychoanalytic psychology (Cohen, 1964; Grof, 1982), few topics can engage scientists from as wide a range of disciplines. This reflects not only the special research value of psychedelics but also the immensity of the challenge involved in understanding them; especially, if the intention is to develop a comprehensive account of how psychedelics affect the brain to alter consciousness. The present article should therefore be read with an acknowledgement that this quest is on-going.

Before we begin, it is necessary to enter some important caveats. Firstly, it needs to be stated that those looking for evidence for the authenticity of aspects of Freudian theory will be left dissatisfied by this article. Categorically, this is not its aim. This challenge requires a thorough review of the phenomenology of relevant altered states of consciousness (e.g., the psychedelic state) and this is something that has been attempted before (Carhart-Harris, 2007; Carhart-Harris and Friston, 2010). Thus, due to space limitations, this article's treatment of the relevant phenomenology is relatively superficial. Instead it places its focus on the system-level mechanics of the psychedelic state as an exemplar of a regressive^8^ style of cognition that can also be observed in REM sleep and early psychosis.

Some proponents of psychoanalysis may feel that this mechanistic approach has little relevance to psychoanalysis in its hermeneutic or interpretative guise. However, the inherent subjectivity of this aspect of psychoanalysis means that it is difficult to see how it can ever significantly impinge on the scientific study of the mind and brain. Indeed, Freud acknowledged that it was his “metapsychology” that had the most to offer science (Freud, 1949), and at least as a first step, this is where psychoanalytic theory (rather than psychoanalytic practice) should look to develop its scientific credibility. Briefly, for readers who are unfamiliar with Freudian metapsychology and wish to understand it better, his original material should be read (e.g., Freud, 1927, 1949; Freud et al., 1957) and the following review articles may be useful (Carhart-Harris et al., 2008; Carhart-Harris and Friston, 2010). For those interested in the rich phenomenology of the psychedelic experience and how this relates to Freudian and/or Jungian descriptions of “the unconscious mind,” the following references may be of interest (Sandison and Whitelaw, 1957; Huxley, 1959; Cohen, 1964; Grof, 1982; Merkur, 1998; Sandison, 2001). Lastly, it is necessary to state that questions related to the safety of scientific research with psychedelics will not be addressed here. However, evidence strongly supports the position that, conducted with appropriate caution, research with psychedelics presents a low risk of harm to study participants (Johnson et al., 2008; Morgan et al., 2010; Carhart-Harris and Nutt, 2010; Studerus et al., 2011; van Amsterdam et al., 2011).

## The pharmacology of psychedelics

Before introducing the focal topic of this paper, i.e., entropy and its relation to key brain imaging parameters, it is important to provide a brief introduction to the pharmacology of psychedelics. By definition, all *classic* psychedelic drugs are agonists at the serotonin 2A receptor (5-HT_2A_R) (Glennon et al., 1984). There is a strong positive correlation between a psychedelic's affinity for the 5-HT_2A_R and its psychedelic potency (Glennon et al., 1984). For example, LSD has a very high affinity for the 5-HT_2A_R and is remarkably potent, being psychoactive in doses as small as 20 μM (Hintzen and Passie, 2010). Blockade of the 5-HT_2A_R with the 5-HT_2A_R antagonist ketanserin, attenuates the principal hallucinogenic effects of psilocybin in humans (Vollenweider et al., 1998). The 5-HT_2A_R is primarily expressed in the cortex (Pazos et al., 1987). In humans, the distribution of 5-HT_2A_Rs is generally high throughout the cortex but is densest in high-level association regions such as the PCC and lowest in the primary motor cortex (Erritzoe et al., 2009; Carhart-Harris et al., 2012a). This may explain why cognition and perception are so markedly affected by psychedelics whereas motor action is generally not. In terms of the cortex's laminar organization, 5-HT_2A_Rs are most densely expressed postsynaptically on the apical dendrites of layer 5 pyramidal neurons (Weber and Andrade, 2010). These large excitatory neurons are the primary source of output from a cortical region, projecting to hierarchically subordinate cortical and subcortical regions (Spruston, 2008). 5-HT_2A_R stimulation depolarizes the host cell, making it more likely to fire (Andrade et al., 2011) and this effect has been demonstrated in layer 5 pyramidal neurons in rodents (Aghajanian and Marek, 1997).

## Functional MRI and MEG studies with psilocybin

Beginning in 2009, our research team embarked on a series of studies with the classic psychedelic, psilocybin (Carhart-Harris et al., 2012a), culminating in a recent MEG study (Muthukumaraswamy et al., 2013). Our first study utilized arterial spin labeling (ASL), an fMRI technique that measures changes in CBF. Specifically, we compared CBF before and after intravenous (i.v.) administration of 2 mg psilocybin and placebo (Carhart-Harris et al., 2012a). The onset of the subjective effects of psilocybin is rapid when it is administered intravenously, commencing within seconds of the end of the infusion (Carhart-Harris et al., 2011). The infusion occurred over 60 s, beginning 6 min into an 18 min resting state scan. Drug-induced changes in CBF were modeled based on psilocybin's rapid pharmacodynamics (Carhart-Harris et al., 2011). Fifteen healthy volunteers were scanned and the results revealed decreased CBF after psilocybin and no increases. The decreases were localized to high-level association cortices, including key regions of the DMN (see Some background on the default mode network (DMN) for an overview of this system) and subcortical hub structures such as the putamen and thalamus (Carhart-Harris et al., 2012a).

These findings were later replicated using the classic BOLD signal of fMRI. Another 15 healthy volunteers were scanned using a similar placebo-controlled design, with 60 s i.v. infusions beginning midway through two separate 12 min eyes-closed resting state scans on different days. Again, only signal decreases were observed after drug infusion. Moreover, the location of the BOLD signal decreases was consistent with the CBF decreases, e.g., in midline cortical nodes of the DMN (Muthukumaraswamy et al., 2013).

**Figure 2:**
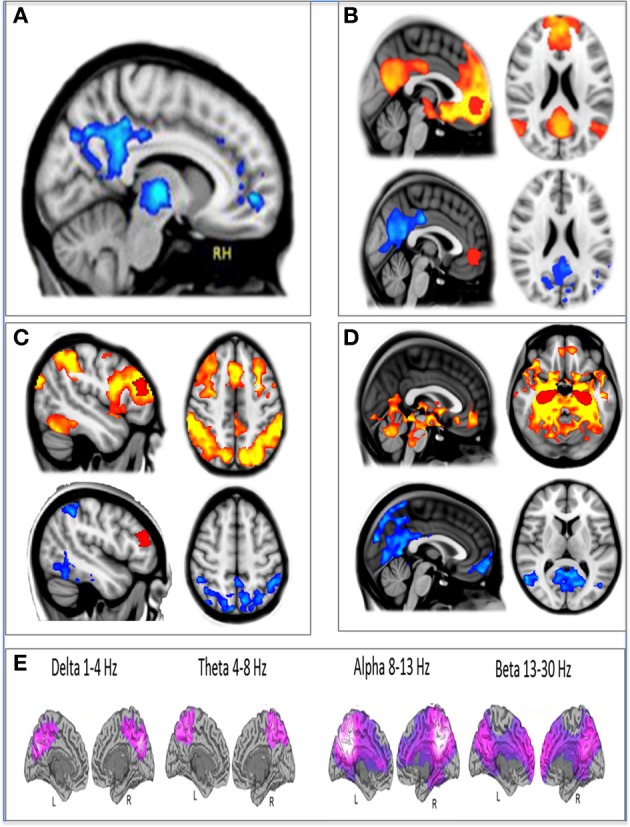
**The effect of psilocybin on fMRI and MEG measures of brain activity. (A)** Decreased CBF post-psilocybin. **(B)** Ventromedial PFC (red) resting state functional connectivity (RSFC) at baseline (top, orange) and decreases post-psilocybin (bottom, blue). **(C)** Dorsolateral PFC (red) RSFC at baseline (top, orange) and decreases post-psilocybin (bottom, blue). **(D)** Hippocampal (red) RSFC at baseline (top, orange) and decreases post-psilocybin (bottom, blue). **(E)** Decreases in oscillatory power (purple) post-psilocybin measured with MEG. All spatial maps were whole-brain cluster corrected *Z* > 2.3. *p* < 0.05.

In addition to modeling changes in the direction of the BOLD signal post-infusion of psilocybin, we also measured changes in brain network integrity using resting-state functional connectivity. Three regions of interest were chosen for separate seed-based resting state functional connectivity (RSFC) analyses: a medial prefrontal cortex (mPFC) seed, a right middle frontal gyrus (mFG) seed, and a bilateral hippocampal seed. Decreased connectivity was observed within the DMN using the mPFC and hippocampal seeds and in a major task-positive network (TPN), the dorsal attention network (DAN), using the mFG seed (Figure 2).

In our third and most recent study, we used MEG to investigate the effects of psilocybin on neural activity. Broadband decreases in oscillatory power were observed after psilocybin, and again, these were localized to association cortices, including key regions of the DMN, such as the PCC (Raichle et al., 2001; Greicius et al., 2003)—see Figure 2.

These studies provide some useful clues about the mechanisms by which psychedelics alter brain function to alter consciousness. They imply that cerebral blood flow, BOLD signal, functional connectivity and oscillatory power are decreased in brain regions that are normally highly metabolically active, functionally connected and synchronous/organized in their activity. These results provided the kernel for our subsequent thinking about increased entropy in the psychedelic state. Although none of the analyses formally measured entropy, they spoke to a general principle that psychedelics alter consciousness by *disorganizing* brain activity.

## Some background on the default mode network

The DMN has become one of the most discussed topics in cognitive neuroscience over the last decade and there are several reasons why it is justified to consider it important (Guldenmund et al., 2012). DMN regions receive more blood flow (Zou et al., 2009) and consume more energy (Raichle and Snyder, 2007) than other brain regions. Indeed, CBF and metabolic rate are approximately 40% higher in the PCC than the average of the rest of the brain (Raichle et al., 2001). The magnitude of the DMN's energy consumption dwarfs the comparatively trivial energy changes induced by stimulus cues (Raichle, 2006, 2010). DMN regions are centers of dense connectivity (Hagmann et al., 2008), implying that they serve as important connector hubs for information integration and routing (van den Heuvel et al., 2012). Consistent with this, a major node of the DMN, the PCC, can be spatially segmented into sub-components that functionally couple to different brain networks (Leech et al., 2012). Similarly, during transient windows of especially high internal coupling (functional connectivity) within the DMN, coupling between the DMN and other brain networks is also markedly increased (de Pasquale et al., 2012). Importantly, this functional centrality of the DMN is not shared by other brain networks (de Pasquale et al., 2012; Braga et al., 2013), implying that, as the highest level of a functional hierarchy (Carhart-Harris and Friston, 2010), it serves as a central *orchestrator* or *conductor* of global brain function. Functionally, the DMN is relatively removed from sensory processing (Sepulcre et al., 2012) and is instead engaged during higher-level, metacognitive operations such as self-reflection (Qin and Northoff, 2011), theory-of-mind (Spreng and Grady, 2010) and mental time-travel (Buckner and Carroll, 2007)—functions which may be exclusive to humans. DMN connectivity increases through development from birth to adulthood (Fair et al., 2008; Gao et al., 2009) and DMN regions have undergone significant evolutionary expansion (Van Essen and Dierker, 2007). Despite our knowledge of these things however, it is poorly understood why the DMN consumes so much of the body's energy (Raichle and Mintun, 2006). This uncertainty regarding the nature of the DMN's disproportionate energy consumption has led to loose analogies being made between it and the *dark energy* of cosmology (Raichle, 2006, 2010). It is consistent with the hypotheses of this paper to suggest that this apparent excess energy of apparently unknown function, residing in the DMN, is in fact the physical counterpart of the narrative-self or ego—much of which is indeed unconscious or implicit.

## Introducing primary consciousness and primary states

This article proposes that states such as the psychedelic state, REM sleep, the onset-phase of psychosis and the dreamy-state of temporal lobe epilepsy are examples of a regressive style of cognition that is qualitatively different to the normal waking consciousness of healthy adult humans. We will refer to this mode of cognition as “primary consciousness”^9^ and the states themselves as “primary states.” To enter a primary state from normal waking consciousness, it is proposed that the brain must undergo a “phase transition” (Zeeman, 1973; Waddington, 1974), just as there must have been a phase-transition in the evolution of human consciousness with the relatively rapid development of the ego and its capacity for metacognition^10^. This implies that the relationship between normal waking consciousness and “primary consciousness” is not perfectly continuous.

Freud was a great admirer of Darwin and made several references to him throughout his work (Freud et al., 1953). Indeed, Freud considered his own hypotheses to be natural deductions from evolutionary theory. He argued that dreaming and psychosis typify a primitive style of thinking that is dominant in human infancy^11^ and dominated the cognition of primordial man^12^, preceding the development of the ego of modern adult humans. Primitive thinking is fundamentally different to the style of cognition possessed by healthy adult humans. This is because in healthy adults, the formation of a mature ego endows the mind with a capacity for metacognition i.e., an ability to reflect on one's own thoughts and behavior (Shimamura, 2000; Fleming et al., 2012).

These ideas form the core of this article's hypotheses. Thus, it is appropriate to clarify them here. A distinction is being made between two fundamentally different styles of cognition, one that is associated with the consciousness of mature adult humans, and another that is a mode of thinking the mind regresses to under certain conditions, e.g., in response to severe stress, psychedelic drugs and in REM sleep. The style of cognition that is dominant in normal waking consciousness will henceforth be referred to as *secondary consciousness*^13^ and the (pre-ego) style of cognition that is associated with primitive states will be referred to as *primary consciousness*. It is acknowledged that these terms have been used before (Edelman, 2004) but their meaning in the present context is largely independent.

Consistent with Karl Friston's free-energy principle (Friston, 2010), this article takes the view that the mind has evolved (via secondary consciousness upheld by the ego) to process the environment as *precisely* as possible by finessing its representations of the world so that *surprise* and *uncertainty* (i.e., entropy) are minimized. This process depends on the ability of the brain to organize into coherent, hierarchically-structured systems (Bassett et al., 2008; Friston, 2010), critically poised between order and disorder (Friston et al., 2012b; Schwartenbeck et al., 2013). In contrast, in primary states, cognition is less meticulous in its sampling of the external world and is instead easily biased by emotion, e.g., wishes and anxieties.

Later we finesse this basic model, arguing that secondary consciousness actually depends on the human brain having developed/evolved a degree of sub-criticality in its functionality, i.e., an extended ability to suppress entropy and thus organize and constrain cognition. It is argued that this entropy-suppressing function of the human brain serves to promote realism, foresight, careful reflection and an ability to recognize and overcome wishful and paranoid fantasies. Equally however, it could be seen as exerting a limiting or narrowing influence on consciousness.

This paper argues that the underlying neurodynamics of primary states are more “entropic” than secondary states i.e., primary states exhibit more pronounced characteristics of criticality and perhaps supercriticality than normal waking consciousness—implying that the latter is slightly sub-critical, if not perfectly critical. Secondary consciousness pays deference to reality by carefully sampling the world and learning from its encounters (Friston, 2010), whereas primary consciousness does this more haphazardly. Mechanistically, whereas the brain strives toward organization and constraint in secondary consciousness, processes are more flexible in primary consciousness. Freud outlined these ideas in his writings on “the reality principle” (Freud, 1927) and they are recast here in a more mechanistic form, tied to modern cognitive neuroscience.

The phenomenon of “magical thinking”^14^ (Frazer, 1900; Subbotskii, 2010; Hutson, 2012) is a potential product of primary consciousness. Magical thinking is a style of cognition in which supernatural interpretations of phenomena are made. Magical thinking is more likely in situations of high uncertainty because there is a greater opportunity for dreaming up explanations that lack an evidence base (Friston, 2010). Wishful beliefs are a classic product of magical thinking because they interpret the world according to what an individual *wants* to be true (in Freudian terms, they adhere to the pleasure principle). Wishful inferences are quick-fixes that reduce uncertainty but via simplistic explanations that satisfy fancies or desires before careful reason. Another example of magical thinking is paranoia; in this case, an individual jumps to negative conclusions about a situation, even in the face of contradictory evidence, because to do so effectively suspends uncertainty while providing some narcissistic satisfaction. The popularity of magical thinking also suggests that there is some enjoyment in uncertainty, perhaps because it promotes imaginative and creative thinking—and that this is associated with positive affect.

In the forthcoming section we discuss the relationship between medial temporal lobe (MTL—i.e., specially the hippocampus and surrounding parahippocampal structures) activity and primary consciousness, highlighting a specific change in activity that may serve as a marker of primary states.

## The medial temporal lobes and primary consciousness

Recording directly from MTL circuits in different altered states presents a significant challenge for cognitive neuroscience, but not one that should deter us from trying to expand its reach into areas of relevance to psychoanalytic theory. Pioneering surgical interventions for epilepsy and Alzheimer's disease (Axmacher et al., 2008, 2010; Fell et al., 2011, 2012; Laxton and Lozano, 2012) are opening up new possibilities for depth recordings, and although it would be a challenge to defend the administration of psychedelics to such patients, recording from MTL circuits in other primary states, such as REM sleep, might be more feasible (Cantero et al., 2003).

Another way to circumvent the problem of recording directly from limbic regions is to use non-invasive imaging with high spatial resolution. We recently used fMRI to investigate the involvement of the MTLs in the mechanism of action of psychedelics, performing a hippocampal functional connectivity analysis using the same psychophysiological interaction (PPI) model used in our previous analyses with psilocybin (Carhart-Harris et al., 2012a). A combined bilateral hippocampal and parahippocampal mask was created on a standard brain and time-series were extracted from these regions for each subject and regressed against their functional data, with the pharmacodynamics of intravenous psilocybin modeled as an interaction term. Remarkably, decreases in functional coupling were observed after psilocybin that were selectively localized to the cortical nodes of the DMN (Figure 2D), entirely consistent with the hypothesis that decreased MTL-DMN coupling underlies phase transitions to primary consciousness.

In a separate analysis, we looked at changes in BOLD signal variance (i.e., amplitude fluctuations) after psilocybin and found significant increases in variance that were almost exclusively localized to the bilateral hippocampi and parahippocampal gyri. This result was important because it reinforced the impression given by the RSFC analysis that under psilocybin, the hippocampi become decoupled from the DMN. However, perhaps even more interestingly, the increase in MTL signal variance was consistent with some early depth electrode work with psychedelics that implicated the MTLs in their mechanism of action. This work is reviewed below.

Human depth recordings involving the insertion of electrodes into structures located deep in the brain (Ramey and O'Doherty, 1960) were not uncommon in the 1950s and 60s. Remarkably, some intracranial recordings were carried out during this period in individuals administered LSD and mescaline (Schwarz et al., 1956; Monroe et al., 1957; Monroe and Heath, 1961). The relevant reports document unusual phasic activities in the MTLs during the drug state that were difficult to detect at the scalp. Moreover, in separate studies, temporal lobectomy was found to abolish the effects of LSD in humans (Serafetinides, 1965) and chimpanzees (Ramey and O'Doherty, 1960) and frontal lobotomy was found to augment them (Keup, 1970). Further support for the involvement of MTLs in the mechanism of action of psychedelics comes from reports of similar phasic limbic activity in other altered states of consciousness that show phenomenological similarities with the psychedelic state, namely: REM sleep, acute psychosis and the so-called “dreamy-state” of temporal lobe epilepsy and electrical stimulation of the MTL (Carhart-Harris, 2007; Carhart-Harris and Friston, 2010). Importantly, these states are all characterized by a particular style of cognition that is fundamentally different from that of normal waking consciousness.

**Figure 3:**
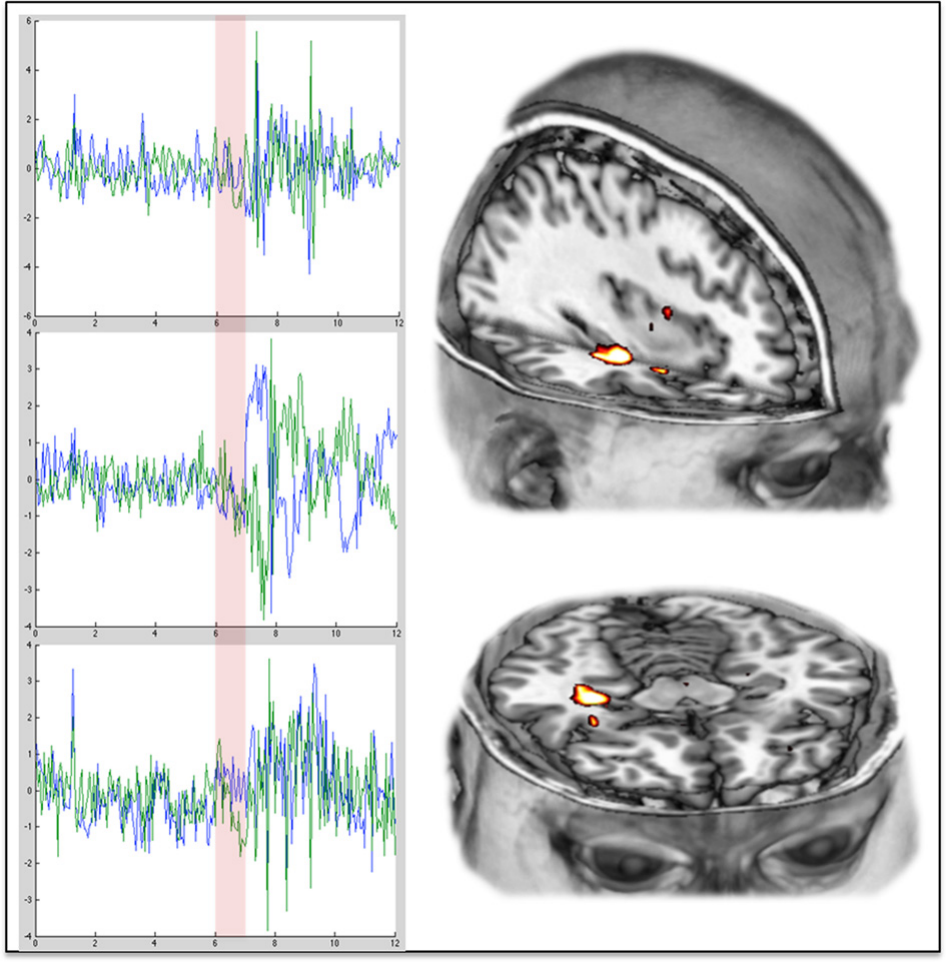
**Increased variance/amplitude fluctuations in the hippocampus post-psilocybin**. The charts on the **left** show the complete time series from the hippocampus (**left** in blue, **right** in green) in 3 different individual subjects during the 12 min scan in which they received psilocybin. The transparent red vertical line indicates the beginning and duration of the 60 s infusion of psilocybin. The images on the **right** show the **right** hippocampal region where the increases in variance were especially marked.

It is proposed here that coupling between the MTLs and the cortical regions of the DMN is necessary for the maintenance of adult normal waking consciousness, with its capacity for metacognition. Moreover, a breakdown in hippocampal-DMN coupling is necessary for a regression to primary consciousness. These hypotheses are motivated by our finding that DMN-hippocampal coupling is decreased under psilocybin (Figure 3D), and while DMN activity becomes desynchronous and therefore disorganized (Figure 3E), the amplitude of BOLD signal fluctuations increases in the hippocampus (Figure 2).

## The default mode network and the ego

**Figure 4:**
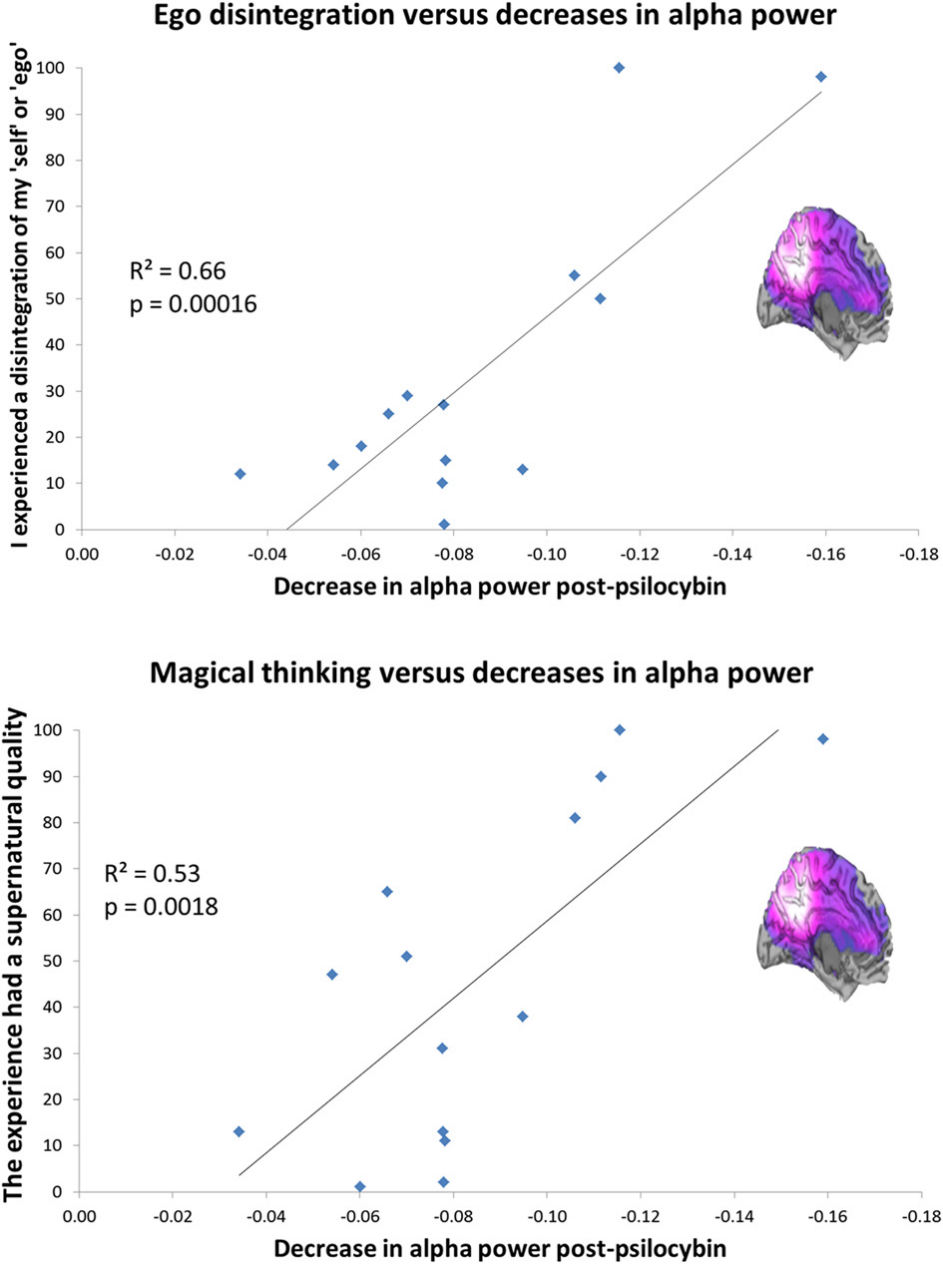
**Decreased PCC alpha power predicts ego-disintegration and magical thinking after psilocybin. Top:** Decreased PCC alpha power *v* ratings of ego-disintegration. **Bottom:** Decreased PCC alpha power vs. ratings of magical/supernatural thinking. Both correlations were significant after correction for multiple comparisons (0.05/23 = 0.002). These charts are derived from data discussed in Muthukumaraswamy et al. (2013).

Layer 5 pyramidal neurons densely express 5-HT_2A_ receptors (Weber and Andrade, 2010). These cells are an important target of psychedelics (Aghajanian and Marek, 1997) and are known to fire with an intrinsic alpha frequency (Silva et al., 1991; Sun and Dan, 2009). Alpha oscillations are thought to be related to temporal framing in perceptual processing (Lorincz et al., 2009; Klimesch et al., 2011) but more intriguingly given the focus of the present article, a positive relationship has been found between self-reflection and alpha power (Knyazev et al., 2011) and alpha synchronization during rest and Blood Oxygen Level Dependent (BOLD) activity in regions of the DMN (Jann et al., 2009). Evidence implicates the DMN in self-reflective and introspective functions (Qin and Northoff, 2011) and the phase of fluctuating activity in the DMN is often inversely correlated (or “anticorrelated”) with fluctuating activity in networks concerned with task-focused attention (task-positive networks, TPNs) (Fox et al., 2005). Like the DMN, alpha oscillations mature developmentally and evolutionarily (Basar and Guntekin, 2009), tempting speculations that these rhythms have developed to reduce “entropy” [i.e., disorder or uncertainty (Ben-Naim, 2008)] by increasing mutual information among neuronal ensembles (Tononi et al., 1994; Basar and Guntekin, 2009). With this in mind, it was remarkable that we recently found a highly significant positive correlation between the magnitude of alpha power *decreases* in the PCC after psilocybin and ratings of the item “I experienced a disintegration of my ‘self’ or ‘ego’.” Scores on this item also correlated positively with decreases in delta, theta, beta, and low gamma power, although alpha explained the most variance (a considerable 66%) see Figure 4. Twenty three subjective items were rated after psilocybin but the one enquiring about ego-disintegration showed the closest relationship with the decreases in alpha power, surviving the conservative Bonferroni correction for multiple comparisons. Interestingly, the only other item that survived correction for multiple comparisons referred to the promotion of magical thinking, i.e., “the experience had a supernatural quality.” It is a central hypothesis of this paper that psychedelics induce a primitive state of consciousness, i.e., “primary consciousness” by relinquishing the ego's usual hold on reality (DMN control on MTL activity).

The organizing influence of alpha applies more generally to oscillatory rhythms in the brain (Salinas and Sejnowski, 2001; Buzsaki and Draguhn, 2004). Harmonics are known to exist between the brain's oscillatory rhythms, with higher frequency oscillations “nested” within lower frequencies (Jensen and Colgin, 2007). For example, intracranial recordings from the ventral PCC in humans revealed a dominant presence of theta oscillations. The phase of these oscillations modulate the amplitude of high-gamma oscillations and the magnitude of this coupling fluctuates at a frequency that is consistent with that of spontaneous BOLD signal fluctuations (i.e., ~0.1 Hz) observed in resting-state networks (RSNs) such as the DMN (Foster and Parvizi, 2012). Theta oscillations are a canonical rhythm of hippocampal circuits, at least in rodents (Buzsaki, 2002), and MTLs are known to be strongly connected to the PCC (Parvizi et al., 2006) and DMN more generally (Kahn et al., 2008). Thus, it is feasible that a function of PCC theta oscillations is to constrain the activity of limbic circuits, which reciprocally input to the PCC. Evidence that MTL activity exerts a *driving* influence on PCC activity comes from a recent report on deep brain stimulation for Alzheimer's disease. Chronic stimulation of the fornix, an important component of hippocampal circuitry, was associated with significantly increased glucose metabolism in the PCC (Laxton et al., 2012).

In summary, interaction between different oscillatory rhythms introduces a structured quality to brain activity (Rumsey and Abbott, 2004), constraining the naturally stochastic firing of individual pyramidal neurons (Rolls and Deco, 2010) and so providing ideal conditions for the emergence of “complexity” (Tononi et al., 1994) or “self-organized criticality” (Jensen, 1998). A key hypothesis of this article is that it is through the development of self-organized activity in the DMN [and concomitant entropy/uncertainty/disorder minimization (Friston, 2010)] that a coherent sense of self or “ego” emerges (Carhart-Harris and Friston, 2010). This process of maturational settling succeeds an earlier state of elevated entropy (primary consciousness) and psychedelic drugs induce a regression to this entropic brain state via the mechanisms outlined above.

**Figure 5:**
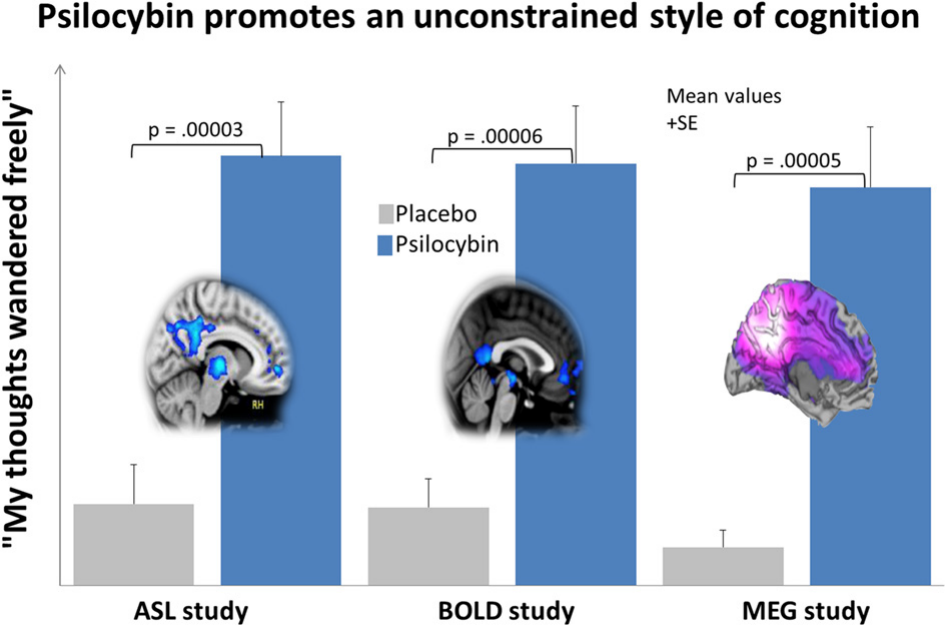
**Psilocybin promotes unconstrained thinking and decreases blood flow, venous oxygenation and oscillatory power in the DMN**. This chart shows the average (+*SE*) ratings for the item “my thoughts wandered freely” in 3 neuroimaging studies, each involving the administration of psilocybin and placebo to 15 healthy volunteers. Ratings were given within 30 min of the end of the relevant resting state scans. This particular item was one of the highest rated items in all 3 studies and nicely communicates the quality of cognition that predominates in the psychedelic state. The brain image on the left displays the mean regional decreases in CBF post-psilocybin in the ASL study; the central image displays the mean regional decreases in BOLD signal post-psilocybin in the BOLD study; and the image on the right displays the mean regional decreases in alpha power post-psilocybin in the MEG study. All images were derived using a whole brain corrected threshold of *p* < 0.05.

With these foundations laid, the following hypotheses can be proposed: (1) coupling within the DMN, and *especially* between the MTL and DMN, is a characteristic of maturational settling that is necessary for secondary consciousness and the development of an integrated sense of self; (2) a relative decoupling within the DMN and specifically between the MTLs and DMN occurs when secondary consciousness abates and there is a reciprocal increase in the influence of primary consciousness; (3) decreased MTL-DMN coupling allows the MTLs to function more independently of the DMN and this can result in unusual MTL activities such as have been recorded with depth electrodes in primary states (see above and Grof, 1982; Bassett et al., 2008; Axmacher et al., 2010) and may have been detected in the BOLD signal amplitude increases in the MTL post-psilocybin (Figure 3); (5) unconstrained/disinhibited/anarchic MTL activity is a principal characteristic of primary states and the occurrence of these activities is consistent with a system at criticality; (6) brain activity in primary consciousness is closer to criticality-proper than it is during normal waking consciousness (which may be slightly sub-critical rather than perfectly critical).

## The DMN, introspection and metacognition

DMN resting-state functional connectivity correlates positively with ratings of internal awareness (Vanhaudenhuyse et al., 2011), depressive rumination (Berman et al., 2011) and trait neuroticism (Adelstein et al., 2011). DMN connectivity increases during mental time-travel (Andrews-Hanna et al., 2010; Martin et al., 2011) and activity in the medial prefrontal node of the DMN is reliably elevated in depression (Farb et al., 2011; Lemogne et al., 2012). These findings strongly implicate the DMN in introspective thought and suggest that hyper activity and connectivity in the DMN is related to a certain style of *concerted* introspection.

To step back, one of the primary hypotheses being developed here is that metacognition, and in particular, the human capacity for self-reflection, is an advanced behavior that rests on self-organized activity in the DMN and between the DMN and the MTLs. Thus, if the DMN is hyper-active and connected in depression, does this imply that mild depression is an evolutionarily advanced state? The phenomenon of “depressive realism” has been recognized for several decades (Dykman et al., 1989; Haaga and Beck, 1995) and sits comfortably with the idea that a primary function of the DMN is to support metacognition (Fleming et al., 2010). The suggestion is that increased DMN activity and connectivity in mild-depression promotes concerted introspection and an especially diligent style of reality-testing. However, what may be gained in mild depression (i.e., accurate reality testing) may be offset by a reciprocal decrease in flexible or divergent thinking (and positive mood).

The proposal that increased DMN activity and connectivity is a key functional correlate of concerted introspection, such as is seen in depression, may seem inconsistent with the association between DMN activity and mind-wandering (Mason et al., 2007) but this is a conceptual problem that can be easily resolved. The positive relationship between increased BOLD signal in the DMN and the frequency of mind-wandering during task-performance (Mason et al., 2007) tells us nothing about the nature or *style* of the cognition in the off-task state, it simply tells us that the mind has drifted off-task. It is known however, that the strength of inverse coupling between activity in the DMN and TPNs is increased when task performance is more consistent (Kelly et al., 2008), implying increased focus and a relative decrease in off-task attentional lapses. DMN-TPN inverse coupling is decreased in patients with attention deficit/hyperactivity disorder (ADHD) (Hoekzema et al., 2013) and increased after administration of the attention-enhancers modafinil (Schmaal et al., 2013) and nicotine (Cole et al., 2010). Thus, it is too simplistic to regard increased BOLD signal in the DMN as a correlate of freely-wandering cognition, and decreased inverse coupling between the DMN and TPN is probably a more informative index of this. As will be discussed later, this point is reinforced by findings that inverse coupling between the DMN and TPNs is decreased under psilocybin, and DMN activity and connectivity is also decreased. This is important because unconstrained, explorative thinking is a hallmark of the psychedelic state (see Figure 5).

In the next section we cite direct evidence for increased entropy in brain networks in psychedelic state and use this to support a general principle: that the transition from normal waking consciousness to primary consciousness is marked by an increase in system entropy.

## Increased network entropy in the psychedelic state

There is an emerging view in cognitive neuroscience that the brain self-organizes under normal conditions into transiently stable spatiotemporal configurations (Sporns et al., 2004; Shanahan, 2010; Deco and Corbetta, 2011; Tagliazucchi et al., 2012) and that this instability is maximal at a point where the global system is critically poised in a transition zone between order and chaos (Tononi et al., 1994; Shanahan, 2010; Deco and Jirsa, 2012; Tagliazucchi et al., 2012). In the present context, the “metastability” (Tognoli and Kelso, 2014) of a brain network is a measure of the variance in the network's intrinsic synchrony over time. That is, if the signal in all of the voxels within a given network deviates little from the network's mean signal, then variance is low, whereas if the signal in voxels fluctuate erratically, then variance is high. Thus, using the data from the BOLD fMRI study with psilocybin, we recently looked at changes in the variance of intra-network synchrony over time in nine canonical resting-state networks (Smith et al., 2009) pre and post placebo and psilocybin. Results revealed significantly increased network variance in high-level association networks after psilocybin but not in sensory specific and motor networks, and there were no changes after placebo. These results imply that activity in high-level networks becomes relatively disorganized under psilocybin, consistent with the entropic brain hypothesis.

**Figure 6:**
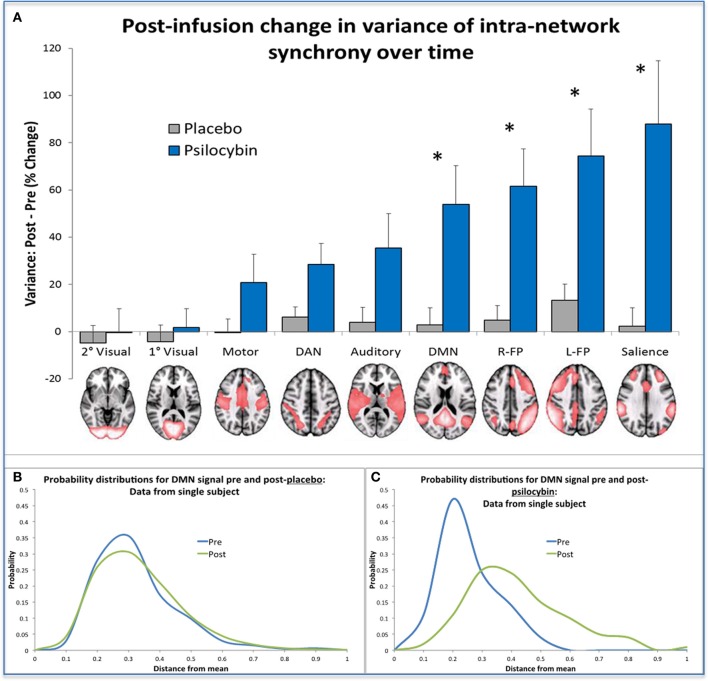
**Changes in network metastability and entropy post-infusion of psilocybin. (A)** This chart displays the mean variance of the internal synchrony of 9 brain networks for the sample of 15 healthy volunteers, as a percentage change post vs. pre-infusion. A post-infusion increase in metastability for a specific network indicates that the mean signal in that network is a poor model of the activity in its constituent voxels, implying that the network is behaving more “chaotically” post-infusion than pre. Bonferonni correction gave a revised statistical threshold of *p* < 0.006 (0.05/9). One-sample (2-tailed) *t*-tests were performed, comparing the % change against zero. The significant networks are labeled with an asterisk. **(B,C)** These probability distributions were derived from data from the same single subject, by discretizing a measure of the internal synchrony of the DMN across time into bins. These bins reflect the distance a data point is from the mean and this gives a probability distribution of the variance of internal synchrony within a network for a given time period (e.g., a 5 min period of scanning). The probability distributions shown in **Chart B** were produced from placebo data where it is clear that prediction of internal network synchrony of the DMN across time is similar before and after infusion (i.e., the blue and green curves). The probability distributions shown in **Chart C** were derived using psilocybin data and here it is evident that following infusion of psilocybin (i.e., the green curve), prediction of internal network synchrony within the DMN is more difficult compared to pre infusion (the blue curve). When the entropy change was calculated for the group, significantly greater increases in entropy were found in the same networks highlighted in **(A)** (post-psilocybin vs. pre) vs. (post-placebo vs. pre).

To translate this result into a formal measure of entropy, we discretized the time course of intra-network synchrony over time into equal sized bins where each time-point could be entered into a bin depending on the variance in the network's synchrony at that time point. Doing this for each network, we built probability distributions of the variance of the intra-network synchrony across time from which we could then calculate the Shannon entropy for each network. Not surprisingly, increased entropy was observed in the networks in which there was increased variance post-psilocybin i.e., the high-level association networks (See Figure 6).

To further assess entropy changes after psilocybin, we took a slightly different approach. Four regions were chosen from a limbic/paralimbic system based on prior knowledge that BOLD signal variance was increased in these regions under psilocybin. The regions included: the left and right hippocampi and the left and right anterior cingulate cortex (ACC). A threshold was set for connection strength such that only connections above a particular strength survived and were therefore said to “exist.” This allowed functional connectivity motifs (connectivity graphs) to be identified at each time point in the time series. With 4 nodes, there were 64 possible connectivity motifs or graphs at any given time point. The results revealed a greater repertoire of motifs under psilocybin than either at baseline or after placebo. Indeed, a number of motifs were exclusive to the psilocybin condition. The entropy of a time series could then calculated by assessing the entropy of a sequence of motifs over a period of time (i.e., how easy/difficult is it to predict a sequence of motifs in a given state?). This is the same procedure one would follow in order to calculate the entropy of a transcribed passage of speech for example (i.e., the likelihood of certain words occurring in a coherent passage is not random, e.g., some words, such as “I,” occur much more often than others). Thus, it was found that the sequence of motifs had significantly greater entropy under psilocybin than at baseline, meaning that a more random sequence of motifs played-out in the psychedelic state. This result implies that it is harder to predict the sequence of connectivity motifs in the psychedelic state because it is more random. This outcome is entirely consistent with the entropic brain hypothesis, which states that brain activity becomes more random and so harder to predict in primary states - of which the psychedelic state is an exemplar.

## Criticality and primary consciousness

The DMN appears to have a consistently high level of activity, e.g., even when the DMN is relatively deactivated during goal-directed cognition, it is still receives more blood flow than elsewhere in the brain (Pfefferbaum et al., 2011). Thus, it can be inferred that one reason why the DMN is so highly and persistently active, is that it receive regular endogenous input from internal drivers. One such driver may be MTL activity (Laxton et al., 2010) and another may be input from brainstem nuclei such as the serotonergic raphe nuclei. Irrespective of what the specific drivers of the DMN are, its enduring presence fits comfortably with the idea that it is the seat of the ego (Carhart-Harris and Friston, 2010), as in healthy waking consciousness, one's sense of self is never far from consciousness:

“Normally, there is nothing of which we are more certain than the feeling of our self, of our own ego.” (Freud, 1930)

So how does the phenomenon of primary consciousness fit in here? The first thing to say is that primary consciousness may be a sub-optimal mode of cognition that has been superseded by a more reality-bound style of thinking, governed by the ego. However, if primary consciousness is a psychological atavism, and the psychedelic state is an exemplar of it, then how does this explain the putative utility of the psychedelic experience e.g., as an adjunct to psychotherapy (Moreno et al., 2006; Grob et al., 2011) and why do some people report being so profoundly affected by such experiences (and often seemingly for the better) (Griffiths et al., 2008; Carhart-Harris and Nutt, 2010; MacLean et al., 2011)?

The phenomenon of depression can help us here. Cognition during an episode of depression is characteristically inflexible; the patient's focus is almost entirely inward and self-critical, and *he/she is unable to remove him/herself from this state* (Holtzheimer and Mayberg, 2011). In the previous section, depressive realism was discussed in relation to hyper activity and connectivity within the DMN; however, in severe depression, cognition cannot be said to be optimal. Depressed patients typically perceive themselves and their world through an unyielding pessimism (Styron, 1992). Depressed patients' cognitive style may become too fixed, such that the patient loses the ability to think and behave in a flexible manner. Underlying this phenomenon may be a decrease in metastability, such that one particular state, e.g., the introspective default-mode, comes to dominate cognition. The aggressive self-critical focus that accompanies a loss or abandonment of object-cathexis in depression (i.e., interest in or focus on objects in the world, such as work and people) quite naturally leads to suicidal thoughts and acts (Carhart-Harris et al., 2008). In consideration of these things, narrow-mindedness is to pessimism what openness (MacLean et al., 2011) is to optimism and strategies that promote the latter may be effective treatments for depression (see MacLean et al., 2011).

**Figure 7:**
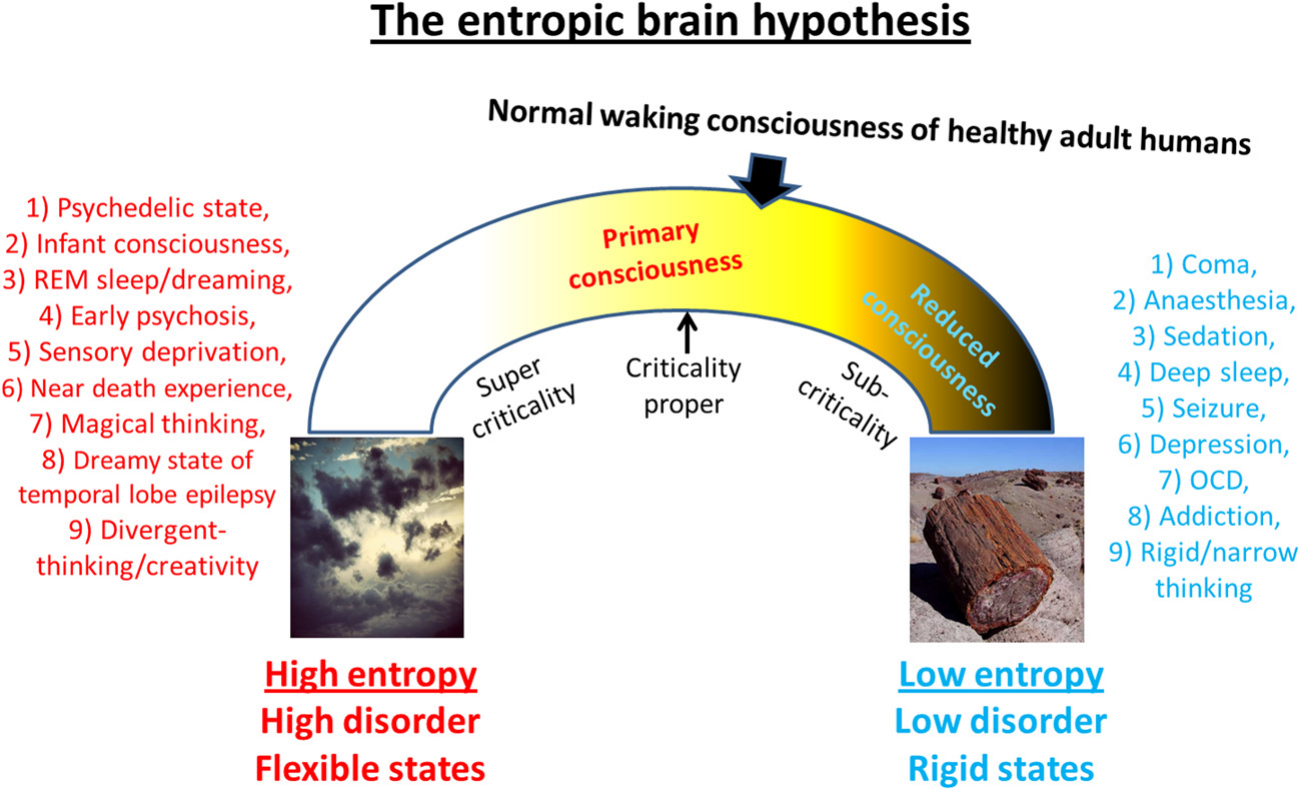
**Spectrum of cognitive states**. This schematic is intended to summarize much of what this paper has tried to communicate. It shows an “inverted u” relationship between entropy and cognition such that too high a value implies high flexibility but high disorder, whereas too low a value implies ordered but inflexible cognition. It is proposed that normal waking consciousness inhabits a position that is close to criticality but slightly sub-critical and primary states move brain activity and associated cognition toward a state of increased system entropy i.e., brain activity becomes more random and cognition becomes more flexible. It is proposed that primary states may actually be closer to criticality proper than secondary consciousness/normal waking consciousness.

This article proposes that primary consciousness rests on more metastable dynamics than secondary consciousness, i.e., brain sub-states are less stable in primary consciousness. Thus, by implication, a broader repertoire of transient states may be visited in primary consciousness because the system is closer to criticality-proper. Moreover, it is the ability of psychedelics to disrupt stereotyped patterns of thought and behavior by disintegrating the patterns of activity upon which they rest that accounts for their therapeutic potential. This principle implies that a brain at criticality may be a “happier” brain. The schematic below (Figure 7) illustrates differences between primary and secondary consciousness. The model describes cognition in adult modern humans as “near critical” but “sub-critical”—meaning that its dynamics are poised in a position between the two extremes of formlessness and petrification where there is an optimal balance between order and flexibility. However, because of maturational settling, the brain in secondary consciousness gravitates toward “order” and thus, the dynamics in this state are more accurately, (slightly) sub-critical. Psychedelics may be therapeutic because they work to normalize pathologically sub-critical styles of thought (such as is seen in depression, OCD or addiction/craving for example) thereby returning the brain to a more critical mode of operating. Indeed, if the principle holds that a critical brain is a happy brain, then it would follow that psychedelics could be used to enhance well-being and divergent thinking, even in already healthy individuals. One negative consequence of this however could be the neglect of accurate reality-testing.

Recent work has indeed supported the notion that brain activity is slightly sub-critical in normal waking consciousness (Priesemann et al., 2013). One reason why it may be advantageous for the brain to operate just below criticality is that by doing so, it can exert better control over the rest of the natural world—most of which *is* critical. This may take the form of suppressing endogenous processes within the brain or interacting with the environment in order to shape it and thereby control it. Indeed, if control is the objective, then it makes sense that the brain should be more ordered than that which it wishes to control.

The idea that the brain is closer to criticality in the psychedelic state than in normal waking consciousness (Figure 7) has some intuitive appeal as some of the signatures of criticality, such as maximum metastability, avalanche phenomena and hypersensitivity to perturbation are consistent with the phenomenology of the psychedelic state. For example, if we consider just one of these: hypersensitivity to perturbation, it is well known that individuals are hypersensitive to environmental perturbations in the psychedelic state, which is why such emphasis is placed on the importance of managing the environment in which the psychedelic experience unfolds (Johnson et al., 2008). Indeed, one explanation for why some people celebrate and romanticize the psychedelic experience and even consider it “sacred” (Schultes, 1980; McKenna, 1992), is that, in terms of criticality, brain activity does actually become more consistent closer with the rest of nature in this state i.e., it moves closer to criticality-proper and so is more in harmony with the rest of nature.

A final speculation that is worth sharing, is that the claim that psychedelics work to lower repression and facilitate access to the psychoanalytic unconscious, may relate to the brain moving out of a sub-critical mode of functioning and into a critical one, enabling transient windows of segregation or modularity to occur (e.g., with “anarchic” MTL activity) because of the breakdown of the system's hierarchical structure. Indeed, repression may depend on the brain operating in a sub-critical mode, since this would constrain consciousness and limit its breadth. Phenomena such as spontaneous personal insights and the complex imagery that often plays out in psychedelic state (Cohen, 1967) and dreaming, may depend on a suspension of repression, enabling cascade-like processes to propagate through the brain [e.g., from the MTLs to the association cortices (Bartolomei et al., 2012)]. Such processes may depend on a reduction of DMN control over MTL activity.

## The therapeutic potential of psychedelics

Many psychiatrists working with psychedelics in the 1950s and 60s expressed great enthusiasm about their therapeutic potential (Crocket et al., 1963; Abramson, 1967; Grinspoon and Bakalar, 1979; Grof, 1980) but there was an unfortunate failure to substantiate these beliefs with properly controlled studies. Subsequent reviews and meta-analyses have suggested an impressive efficacy, especially in relation to the use of LSD in the treatment of alcohol dependence (Mangini, 1998; Dyck, 2005; Krebs and Johansen, 2012) and modern trials have lent some support to this sentiment (Moreno et al., 2006; Grob et al., 2011). For example, a single high dose of psilocybin produced profound existential experiences in healthy volunteers that had a lasting beneficial impact on subjective well-being (Griffiths et al., 2006, 2008) and a moderate single dose of psilocybin administered to patients with advanced-stage cancer significantly reduced anxiety and depression scores for months after the acute experience (Grob et al., 2011). In another study, symptoms of obsessive compulsive disorder (OCD) were significantly reduced after psilocybin (Moreno et al., 2006). Supplementing these controlled studies, we surveyed over 500 recreational drug users, and found that 67% of LSD users and 60% of psilocybin users claimed that use of these drugs had produced long-term positive effects on their sense of well-being (Carhart-Harris and Nutt, 2010), consistent with the results of the aforementioned controlled studies (Griffiths et al., 2006, 2011). To place this in a context, only 6% of alcohol users claimed such improvements from alcohol use (Carhart-Harris and Nutt, 2010). One of the most remarkable properties of psychedelics is their potential to have a lasting impact on personality and outlook (McGlothlin and Arnold, 1971; Studerus et al., 2011). Personality traits are known to be relatively fixed by adulthood (Costa and McCrae, 1997; McCrae and Costa, 1997), however, the personality trait “openness” was found to be significantly increased over 14 months after a single controlled administration of psilocybin (MacLean et al., 2011). Moreover, neuroimaging studies (Carhart-Harris et al., 2012a) have found decreased activity and connectivity after psilocybin in brain regions (e.g., the mPFC) and networks (e.g., the DMN) that are over-engaged in depression (Greicius et al., 2007; Berman et al., 2011) but normalized by a range of effective treatments (Goldapple et al., 2004; Mayberg et al., 2005; Kennedy et al., 2007; Deakin et al., 2008).

Classic psychedelics are all agonists at the serotonin 2A receptor (Glennon et al., 1984; Vollenweider et al., 1998) and 5-HT_2A_R antagonism blocks the positive mood effects of psilocybin (Kometer et al., 2012) and MDMA (van Wel et al., 2012). 5-HT_2A_R expression is upregulated in depression (Bhagwagar et al., 2006) likely because of low synaptic 5-HT (Cahir et al., 2007). Positron emission tomography (PET) studies in humans found positive correlations between 5-HT_2A_R binding and trait neuroticism (Frokjaer et al., 2008) and pessimism (Meyer et al., 2003). This may imply that 5-HT_2A_R upregulation, due to low synaptic 5-HT, reflects a state of chronically deficient post-synaptic 5-HT_2A_R stimulation that contributes to inflexible patterns of (negative) thought such as are seen in depression. 5-HT_2A_R-stimulation may therefore work to reverse this, effectively “lubricating” cognition.

Given our knowledge of the biological effects psychedelics, a comprehensive model can be presented in which psychedelics: (1) stimulate the 5-HT_2A_ receptor (Glennon et al., 1984), (2) depolarize deep-layer pyramidal neurons (Andrade, 2011), (3) desynchronize cortical activity, (4) “disintegrate” brain networks (Carhart-Harris et al., 2012a), (5) increase network metastability and (6) increase the repertoire of connectivity motifs within a limbic/paralimbic network. The net effect of these processes is an increase in system entropy (formally reflected in points 5 and 6) as the system enters criticality-proper.

Thus, in summary, it is hypothesized that there is a basic mechanism by which psychedelics can be helpful in psychiatry, whether they be used to treat depression, OCD (Moreno et al., 2006) or addiction (Krebs and Johansen, 2012). Specifically, it is proposed that psychedelics work by dismantling reinforced patterns of negative thought and behavior by breaking down the stable spatiotemporal patterns of brain activity upon which they rest. An important caveat however, is that in order for this process to be beneficial, the drug-induced transitions to, and the return from primary consciousness, must be mediated by appropriate therapeutic care (Johnson et al., 2008). Moving the brain out of sub-critical modes and into unfamiliar terrain may present some risks (e.g., loss of contact with reality and persistent magical/delusional thinking) if not properly managed (Johnson et al., 2008).

## The spiritual experience and primary consciousness

“If we consider contemporary accounts of the mystical consciousness, we can see that the individuality, the “I,” disappears and is in a sense “annihilated.” (Stace, 1961)

In the psychology of religion, one of the most remarkable findings has been that it is possible, by way of a single high dose of psilocybin, to reliably induce profound spiritual experiences in healthy volunteers that are effectively indistinguishable from spontaneously-occurring spiritual experiences (Griffiths et al., 2006). Perhaps this finding should not be so surprising, psilocybin containing mushrooms have been used for centuries in shamanic “healing” ceremonies (Hofmann, 1980), and in a famous study in the 1960s, high-dose psilocybin was administered to theology students partaking in a religious service on Good Friday and emphatic spiritual experiences were reported (Doblin, 1991). The so-called “entheogenic” (generating the divine) properties of psilocybin appear to be shared by the other classic psychedelics such as LSD and DMT but not the “psychedelic-like” compounds, MDMA and cannabis (Carhart-Harris and Nutt, 2010; Lyvers and Meester, 2012). It is intriguing that entheogenic properties appear to be specific to 5-HT_2A_R agonist classic psychedelics and this suggests a key role for this receptor in their genesis.

In William James' famous lectures on the psychology of religion he proposed that spiritual experiences depend on the emergence of what he referred to as the “subconscious” or “subliminal” mind into consciousness (James, 1968). Referring to what psychoanalysis calls “the unconscious.” James said: “*[T]his is obviously the larger part of each of us, for it is the abode of everything that is latent and the reservoir of everything that passes unrecorded or unobserved… It is the source of our dreams… In it arise whatever mystical experiences we may have… It is also the fountain-head of much that feeds our religion. In persons deep in the religious life—and this is my conclusion—the door into this region seems unusually wide open*.” (James, 1968).

James' ideas are consistent with those of Carl Jung; however, Jung extended them, arguing that the unconscious hosts the psychological remnants of our phylogenetic ancestry. In dreams, psychosis and other altered states, archetypal themes shaped by human history emerge into consciousness (Jung, 1982a). Jung's account of the “collective” unconscious fits comfortably with the phenomenology of the psychedelic experience. Archetypal themes feature heavily in user “trip reports” (Masters and Houston, 1966; Shanon, 2002), as they do in religious iconography. For Jung, religion is a manifestation of the collective unconscious, expressed in a symbolic and ritual form: “*The brain is inherited from its ancestors; it is the deposit of the psychic functioning of the whole human race. In the brain, the instincts are preformed, and so are the primordial images which have always been the basis of man's thinking—the whole treasure-house of mythological motifs… Religious symbols have a distinctly “revelatory” character; they are usually spontaneous products of unconscious psychic activity… they have developed, plant-like, as natural manifestations of the human psyche*.” (Jung, 1982b).

Jung's ideas offer an appealing explanation for the content of religious experiences, as well as the content of high-dose psychedelic experiences; however, a more systematic treatise on the spiritual experience was provided by Walter Stace in 1960 (Stace, 1961). Stace's work is particularly useful because his ideas resonate with the findings of recent neuroimaging studies relevant to the neurobiology of spiritual experiences. Based on a thorough review of first-person accounts derived from individuals from a variety of different faiths, Stace identified seven universal components of the spiritual experience: 1) diminished spatial and temporal awareness, 2) diminished subjectivity (equivalent to increased objectivity), 3) feelings of profound joy and peace, 4) a sense of divinity, 5) paradoxicality (where two opposing things appear to be true), 6) ineffability (the difficulty of expressing the experience in words) and 7) a sense of oneness with the world, otherwise known as “the unitive experience.”

Importantly, in Stace's synopsis, he identified the unitive experience as the core characteristic of the spiritual experience. Freud referred to the same phenomenon as the “oceanic state” (Freud, 1930). Stace explained that in profound spiritual experiences the complex *multiplicity* of normal consciousness collapses into a simpler state where a sense of an all-encompassing unity or “oneness” with others, the world and/or “God” is felt. He emphasized that there is a collapse in the most fundamental dualities of consciousness (i.e., *self* vs. *other*, *subject* vs. *object* and *internal* vs. *external*) in the unitive state. Moreover, he also showed that reports of unitary consciousness are consistent throughout the different religions—emphasizing its universality and cultural independence (Stace, 1961).

Freud had some important things to say about the unitive state that are directly relevant to the entropic brain hypothesis. For example, when discussing his friend's description of an “oceanic feeling” when in religious practice, Freud says: “*Pathology has made us acquainted with a great number of states in which the boundary lines between the ego and the external world become uncertain… Further reflection tells us that the adult's ego-feeling cannot have been the same from the beginning. It must have gone through a process of development… (For example,) an infant at the breast does not as yet distinguish his ego from the external world; he gradually learns to do so. Our present ego-feeling is therefore only a shrunken residue of a much more inclusive—indeed, an all-embracing feeling, which (early in development] corresponded to a more intimate bond between the ego and the world. If we assume that there are many people in whose mental life this primary ego-feeling has persisted to a greater or less degree, it would exist in them side by side with the narrower and more sharply demarcated ego feeling of maturity, like a counterpart to it. In that case, the ideational contents appropriate to it would be precisely those of limitlessness and of a bond with the universe—the same ideas with which my friend elucidated the “oceanic feeling.””* (Freud, 1930).

Thus, Stace and Freud's descriptions of the spiritual experience are entirely consistent with the view of the primary state as being regressive. Moreover, they are also consistent with view that the human brain developed through ontogeny and phylogeny to minimize disorder/uncertainty (Friston, 2010). In the schematic presented in Figure 7, primary consciousness is depicted as being more supercritical than normal waking consciousness. Indeed, at the extreme end of supercriticality is maximum entropy, which is equivalent to formlessness or complete disorder. Formerly, there is no difference between entropy in this thermodynamic sense (depicted as complete disorder) and entropy in the information theory sense, where there is maximum uncertainty about the system - because there is no order on which to base any predictions.

## The system mechanics of primary states

Extending this, the mechanics underlying the onset of true primary states (for which the spiritual experiences is an example) can be viewed in relation to the second law of thermodynamics. Explicitly, in the absence of a regular driving input, the system (i.e., self-organized brain activity) will inevitably degrade or collapse toward formlessness or maximum entropy. The interesting question that follows therefore is: *what is the driving input that ceases in primary states*? This paper proposes that regular MTL activity is a crucial driver of the DMN. Although, interestingly, there is also evidence that the usual clock-like firing of serotonin neurons in the dorsal raphe nuclei completely ceases in both the psychedelic state (Aghajanian et al., 1968; Aghajanian and Vandermaelen, 1982) and REM sleep (Trulson and Jacobs, 1979) and there is some indirect evidence that the DMN may be (at least partially) a serotonergic system coupled to dorsal raphe activity (Zhou et al., 2010).

During secondary consciousness, the brain can enter a *multiplicity* of different states and microstates (Tononi, 2010) but due to “winner-takes all,” or more strictly, “winnerless” (Rabinovich et al., 2001) competition between states [“winnerless” because critical instability or metastability dictates that a state's “victory” is transient (Friston et al., 2012b)] the global system only ever entertains *one* winning state at any one time (Baars, 2005). However, according to the entropic brain hypothesis, in primary states, the potential multiplicity of possible states is not obliterated but rather extended because the *selectivity* and *conspicuity* of a winning state is reduced, and so more transient states may be visited. In dynamical terms, this would be expressed as attractor basins or valleys (defining transient states) becoming shallower in primary states, i.e., the attractor landscape is flattened in primary states.

Conversely, in depression, OCD and addiction, specific states (e.g., the default-mode in depression) may be frequented more regularly than others—and this may be observed as certain mental states (e.g., introspection in depression or craving in addiction) or behaviors (e.g., compulsive acts in OCD) being habitually revisited in a stereotyped fashion. Moreover, these states may be relatively stable i.e., their basins of attraction are relatively steep since the patterns of activity upon which they rest have become entrenched. In such scenarios, uncertainty about the system is minimized because we know that it possesses a particular character. It is intriguing to consider therefore that disorders such as depression, OCD and addiction could be functional in some sense, perhaps working to resist a more catastrophic collapse to primary consciousness (with the onset of a psychotic episode for example) by reinforcing stable patterns of activity.

The following example may help to illustrate what is meant by competition between conscious states—and the loss of it in primary consciousness. Functional brain imaging has identified distinct brain networks that subserve distinct psychological functions. For example, the DMN is associated with introspective thought (Andrews-Hanna et al., 2010) and a dorsal frontoparietal attention network (DAN) is associated with visuospatial attention (Corbetta et al., 1998; Fox et al., 2006) and is a classic example of a “task positive network” (TPN)—i.e., a network of regions that are consistently activated during goal-directed cognition. If the brain was to be sampled during a primary state (such as a psychedelic state) we would predict that the rules that normally apply to normal waking consciousness will become less robust. Indeed, we recently found this to be so when analysing the degree of orthogonality or “anti-correlation” between the DMN and TPN post-psilocybin. Post-drug there was a significant reduction in the DMN-TPN anticorrelation, consistent with these networks becoming less different or more similar (i.e., a flattening of the attractor landscape). The same decrease in DMN-TPN anticorrelation has been found in experienced meditators during rest (Brewer et al., 2011) and meditation (Froeliger et al., 2012). Moreover, decreased DMN-TPN inverse coupling is especially marked during a particular style of meditation referred to as “non-dual awareness” (Josipovic et al., 2011). This is interesting because this style of meditation promotes the same collapse of dualities that was identified by Stace (and Freud) as constituting the core of the spiritual experience. The DMN is closely associated with self-reflection, subjectivity and introspection, and task positive networks are associated with the inverse of these things, i.e., focus-on and scrutiny of the external world (Raichle et al., 2001). Thus, it follows that DMN and TPN activity *must* be competitive or orthogonal in order to avoid confusion over what constitutes *self*, *subject* and *internal* on the one hand, and *other*, *object* and *external* on the other. It is important to highlight that disturbance in one's sense of self, and particularly one's sense of existing apart from one's environment, is a hallmark of the spiritual (Stace, 1961) and psychedelic experience (Carhart-Harris et al., 2012b). Moreover, as in the psychedelic state (Carhart-Harris et al., 2012a; Carhart-Harris et al., 2012b), a number of studies have found decreased DMN activity (Farb et al., 2007; Brewer et al., 2011; Hasenkamp et al., 2012) as well as decreased DMN-TPN inverse coupling in meditation (Brewer et al., 2011; Josipovic et al., 2011; Froeliger et al., 2012).

The contravention or corruption of important rules about brain organization may explain the sense of confusion and uncertainty that accompanies a transition from secondary to primary consciousness. In the information theoretical sense, “uncertainty” is a synonym for entropy (Friston, 2010; Ben-Naim, 2012)—and disorder and uncertainty are effectively equivalents. Entropy in information theory is reflected in the shape of a probability distribution (Ben-Naim, 2012), i.e., we have less confidence (or more uncertainty) about something when the distribution is broader or more evenly spread. This is because it is more difficult to predict what the outcome of an individual sampling trial would be because the system behaves relatively randomly (Ben-Naim, 2012). Conversely, a probability distribution with a sharp peak would reflect a well-ordered system or high-precision, confidence or assuredness (Friston, 2010). In the specific context of the DMN and the psychedelic state, just as there is increased variance in parameters defining the DMN (e.g., coupling between the nodes of the DMN or rhythmic alpha oscillations in the PCC), so there is uncertainty about ones sense of self—typically described as “ego-disintegration.” Thus, according to the entropic brain hypothesis, just as normally robust principles about the brain lose definition in primary states, so confidence is lost in “how the world is” and “who one is” as a personality.

**Figure 8:**
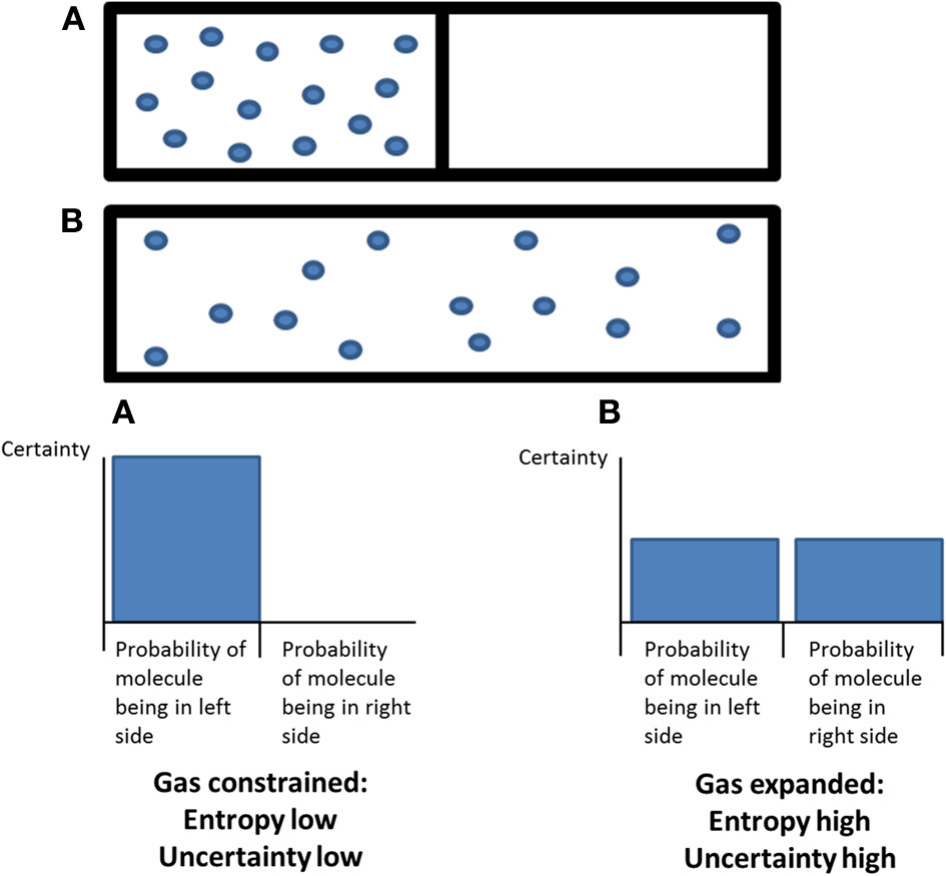
**Gas expansion post-release of constraints as a metaphor for increased entropy in primary states. (A)** Entropy is low while the gas is constrained. **(B)** Entropy increases once constraints are released. In an information theoretical sense, entropy/uncertainty is increased post-expansion because it is more difficult to predict the spatial location of a single molecule. In primary vs. secondary states, it is hypothesized that the biological parameters known to define key brain states (e.g., the default-mode) become more variant or less predictable, thus causing the subject to become less certain in themselves and their experience of the world.

In addition to the word “uncertainty,” other terms that have been used as synonyms for entropy include: “freedom,” “disorder” and “expansion.” The example of a gas expanding post release of constraints is often used as a metaphor to help explain what is meant by entropy increasing [e.g., in relation to the second law of thermodynamics (Ben-Naim, 2012)] See Figure 8. In the information theoretical sense, entropy/uncertainty increases as the gas expands because with greater expansion, it is more difficult to predict the spatial location of a single molecule. It is probably not coincidental that these physical principles resonate with popular descriptions of the psychedelic experience (Huxley, 1959; Bowers and Freedman, 1966; Masters and Houston, 1972; Grinspoon and Bakalar, 1981; Merkur, 1998). For example, the term “consciousness-expansion” is often used to describe the psychedelic experience—and this may be an inadvertent reference to increased system entropy in the psychedelic state.

## Developing the construct validity of primary consciousness

To develop the construct validity of primary consciousness, it will be necessary to show that the identified parameters of primary consciousness have high *internal* and *convergent* validity (i.e., properties of primary consciousness must be shown to exist in a range of different primary states) and sufficient *discriminant* validity (i.e., these properties must be shown to be sufficiently *specific* to primary states, i.e., to be absent in non-primary states). To assist this undertaking, it will be important to optimize subjective rating scales designed for assessing primary consciousness. For example, first it will be necessary to identify key experiences that are considered characteristic of primary states (i.e., visual analog scale items such as “my imagination was extremely vivid” or “the experience had a dreamlike quality” that can be rated during or after a candidate primary state) and then it will be important to test whether different candidate primary states (e.g., dreaming, onset-phase psychosis, the near death experience, the sensory-deprived state and the psychedelic drug state) score highly on these items—and that candidate non-primary states (e.g., normal waking consciousness, the anaesthetized or sedated state, and the depressed state) do not. This will enable the convergent and discriminant validity of these measures to be tested and developed. Similarly, by identifying neurobiological characteristics hypothesized to be essential to primary states (e.g., decreased DMN-MTL coupling, disinhibited MTL activity, decreased DMN-TPN anti-correlation, decreased alpha power in the PCC and increased metastability in resting-state networks), it will be important to determine those that most *reliably* and *specifically* identify primary over non-primary states.

As discussed in The research value of psychedelics, psychedelic drugs are especially useful tools for studying primary states as they allow for primary consciousness to be “switched on” with a relatively high degree of experimental control (e.g., with intravenous infusion of a classic psychedelic). Hypotheses about the neurobiological character of primary states can therefore be effectively tested by psychedelic drugs. However, in order to test and develop the generalizability of these hypotheses, research with alternative primary states are required. For example, it would be interesting to carry out simultaneous fMRI-EEG or MEG work with a focus on REM sleep, or to study patients exhibiting early-phase psychotic symptoms with these techniques. Longitudinal analyses looking at brain maturation would also be relevant, where infant consciousness is hypothesized to be reflective of primary consciousness.

## Developing the construct validity of secondary consciousness and the ego

As outlined above, a key distinction between the primary and secondary modes of cognition is that secondary consciousness pays deference to reality and diligently seeks to represent the world as *precisely* as possible, whereas primary consciousness is less firmly anchored to reality and is easily misled by simple explanations motivated by wishes and fears. One way this distinction could be tested would be to utilize a measure of metacognitive accuracy (Fleming et al., 2010). As outlined above, metacognition, and specifically the ability to reflect upon one's own introspection, is a particularly advanced behavior associated with the DMN (Fleming et al., 2010). For example, a behavioral paradigm could be designed that requires a participant's friend to rate the participant's personality, e.g., using a standard personality inventory. Then, during scanning, the participant could be asked to predict their friend's ratings—and crucially, to provide an additional confidence rating for their own predictions. This could be done under a psychedelic drug and under placebo in a within-subjects design with 2 different friends for each condition, counterbalanced for key factors (e.g., familiarity, intimacy, fondness, duration of relationship etc). This task would provide a behavioral index of a high-level metacognitive function associated with the ego (theory-of-mind). The hypothesis would be that participants would be *less confident* in their predictions of their friend's ratings post-psilocybin and that the accuracy of their predictions would also be compromised. Biologically, one would hypothesize decreased within-DMN coupling during the prediction process and a reduction in induced alpha-oscillations in the PCC.

## Conclusions

This article has argued that scientific research with psychedelic drugs can have a revitalizing effect on psychoanalysis and an informing influence on mainstream psychology and psychiatry. Rather than discuss the content and interpretation of psychoanalytically-relevant material, we have adopted a mechanistic approach, in keeping with the mainstream cognitive neuroscience. This article proposes that a distinction can be made between two fundamentally different modes of cognition: primary and secondary consciousness. Primary consciousness is associated with unconstrained cognition and less ordered (higher-entropy) neurodynamics, whereas secondary consciousness is associated with constrained cognition and more ordered neurodynamics (i.e., that strikes an evolutionarily advantageous balance between order and disorder - that may or more not be perfectly “critical”). It is hoped that this mechanistic model will help catalyze a synthesis between psychoanalytic theory and cognitive neuroscience that can be mutually beneficial to both disciplines.

It is a fair criticism of this paper that it has given insufficient consideration to the phenomenological content of the relevant altered states of consciousness, and to the specifics of Freudian theory, and so by neglecting this, has failed to present a sufficiently compelling case that these states have anything to do with psychoanalytic theory. To some extent, this charge can be conceded; however, as outlined in the introduction, the intention of this paper was to develop a mechanistic account of altered states of consciousness based on the quantity of entropy, and this task has demanded a substantial amount of space. A more thorough discussion of the phenomenology of primary states is required to develop the case that they show characteristics that are consistent with Freudian accounts of “the unconscious” or “Id.” The reader should be made aware however, that this has been attempted before (Carhart-Harris, 2007; Carhart-Harris and Friston, 2010).

To conclude, it is perhaps not surprising that with only dreaming and psychosis at its disposal, psychoanalysis has failed to convince the scientific community that the psychoanalytic unconscious exists (Hassin et al., 2005). From a neuroscientific perspective, dreaming and psychosis are notoriously difficult to study. The occurrence of dreaming in sleep impedes experimental control and psychosis is an especially complex and variegated phenomenon. However, for those brave enough to embrace it, research with psychedelics could herald the beginning of a new scientifically informed-psychoanalysis that has the potential to influence modern psychology and psychiatry. The unique scientific value of psychedelics rests in their capacity to make consciously accessible that which is latent in the mind. This paper takes the position that mainstream psychology and psychiatry have underappreciated the depth of the human mind by neglecting schools of thought that posit the existence an unconscious mind. Indeed, psychedelics' greatest value may be as a remedy for ignorance of the unconscious mind.

“He who would fathom the psyche must not confuse it with consciousness, else he veils from his own sight the object he wishes to explore.” (Jung, 1961)

“Man's worst sin is unconsciousness.” (Jung, 1969)

### Conflict of interest statement

The authors declare that the research was conducted in the absence of any commercial or financial relationships that could be construed as a potential conflict of interest.

## Footnotes

^1^Entropy in its purest information theoretical sense is a dimensionless quantity that is used for measuring uncertainty or ignorance about the state of a system. By implication, entropy/uncertainty is greater the more random a system is. Thus, entropy is most strictly a measure of uncertainty but it also reflects the degree of randomness or disorder in a system (Ben-Naim, 2012).

^2^The free energy principle is an extension of predictive coding (Dayan et al., 1995) and tries to explain how biological systems, such as the brain, maintain their order by developing inferences and behaviors that serve to minimize surprise and uncertainty. Free-energy is formally related to entropy in the information theoretical sense, where entropy (uncertainty) is the average of free-energy (surprise) (Friston, 2010). See also (Friston et al., 2012a) for a philosophical discussion of free-energy.

^3^Self-organized criticality (SOC) is a property of certain systems that gravitate toward a ‘critical’ point in a transition zone between order and disorder. Critical systems display certain characteristics such as power-law scaling (Bak et al., 1987). SOC is typically observed in slowly driven non-equilibrium systems, with many units that interact in a non-linear fashion (Bak et al., 1987; Jensen, 1998).

^4^The ego can be defined as a sensation of possessing an integrated and immutable identity, i.e., “this is me” or “I am like this.” It is equivalent therefore with one's sense of self. In psychoanalytical theory however, the ego is also a system which works in concert with and against other processes in the brain to determine the quality of consciousness. It is worth noting that Freud's term for the ego was ‘the I’ and it was only in the standard translation from German that the term “the ego” became associated with Freud (1927). In everyday (lay) usage, “ego” has become synonymous with exaggerated self-confidence or an inflated ego/sense of self.

^5^A fully-developed, adult ego or sense of self.

^6^A network of functionally and structurally connected brain regions that show high spontaneous or “on-going” metabolism yet a relative deactivation during goal-directed cognition (Raichle et al., 2001).

^7^Functional connectivity is defined as temporal correlations between spatially distinct neurophysiological events (Karl Friston). Resting-state refers to task-free, unconstrained experimental conditions (typically sitting or lying still with eyes closed).

^8^The quality of returning to an earlier state of development.

^9^A regressive, pre-ego style of consciousness characterized by unconstrained brain dynamics and cognition. The psychedelic state is an exemplar of primary consciousness and REM sleep and early psychosis are others.

^10^A cognitive capacity unique to adult humans and dependent on the formation of a mature ego. Metacognition means “cognition about cognition.” Examples include: self-reflection, theory-of-mind and mental time-travel. (Fleming et al., 2012).

^11^The term “infancy” is used here in an extended sense to mean the period from birth to two years of age.

^12^The earliest humans, i.e., archaic homo-sapiens.

^13^The style of consciousness of healthy, adult, modern humans during waking. Secondary consciousness is “constrained” relative to primary consciousness, both in a mechanistic and qualitative sense.

^14^A style of cognition in which causal relationships between phenomena are assumed despite an absence of supportive scientific evidence. Examples include superstition, and metaphysical beliefs. Crucially, such beliefs usually honor an emotional sentiment such as a wish (or fear) that something is true.
